# Obesity, oxidative DNA damage and vitamin D as predictors of genomic instability in children and adolescents

**DOI:** 10.1038/s41366-021-00879-2

**Published:** 2021-06-22

**Authors:** Moonisah Usman, Maria Woloshynowych, Jessica Carrilho Britto, Ivona Bilkevic, Bethany Glassar, Simon Chapman, Martha E. Ford-Adams, Ashish Desai, Murray Bain, Ihab Tewfik, Emanuela V. Volpi

**Affiliations:** 1 School of Life Sciences, University of Westminster, London, UK; 2 School of Social Sciences, University of Westminster, London, UK; 3 Department of Child Health, King’s College Hospital, London, UK; 4 Department of Child Health, King’s College Hospital, London, UK; 5 Department of Paediatric Surgery, Royal London Hospital, London, UK; 6 Department of Paediatric Endocrinology, St George’s University Hospital, London, UK

**Keywords:** Obesity, Risk factors, Cancer

## Abstract

**Background/objectives:**

Epidemiological evidence indicates obesity in childhood and adolescence to be an independent risk factor for cancer and premature mortality in adulthood. Pathological implications from excess adiposity may begin early in life. Obesity is concurrent with a state of chronic inflammation, a well-known aetiological factor for DNA damage. In addition, obesity has been associated with micro-nutritional deficiencies. Vitamin D has attracted attention for its anti-inflammatory properties and role in genomic integrity and stability. The aim of this study was to determine a novel approach for predicting genomic instability via the combined assessment of adiposity, DNA damage, systemic inflammation, and vitamin D status.

**Subjects/methods:**

We carried out a cross-sectional study with 132 participants, aged 10–18, recruited from schools and paediatric obesity clinics in London. Anthropometric assessments included BMI Z-score, waist and hip circumference, and body fat percentage via bioelectrical impedance. Inflammation and vitamin D levels in saliva were assessed by enzyme-linked immunosorbent assay. Oxidative DNA damage was determined via quantification of 8-hydroxy-2′-deoxyguanosine in urine. Exfoliated cells from the oral cavity were scored for genomic instability via the buccal cytome assay.

**Results:**

As expected, comparisons between participants with obesity and normal range BMI showed significant differences in anthropometric measures (*p* < 0.001). Significant differences were also observed in some measures of genomic instability (*p* < 0.001). When examining relationships between variables for all participants, markers of adiposity positively correlated with acquired oxidative DNA damage (*p* < 0.01) and genomic instability (*p* < 0.001), and negatively correlated with vitamin D (*p* < 0.01). Multiple regression analyses identified obesity (*p* < 0.001), vitamin D (*p* < 0.001), and oxidative DNA damage (*p* < 0.05) as the three significant predictors of genomic instability.

**Conclusions:**

Obesity, oxidative DNA damage, and vitamin D deficiency are significant predictors of genomic instability. Non-invasive biomonitoring and predictive modelling of genomic instability in young patients with obesity may contribute to the prioritisation and severity of clinical intervention measures.

## Introduction

There is growing epidemiological evidence that indicates obesity in childhood and adolescence to be an independent risk factor for cancer and premature mortality in adulthood. An increased BMI in childhood has been associated with an increased risk of developing lung, colorectal, kidney, cervical, ovarian and smoking-related cancers later in life [1–4]. Furthermore, a recent meta-analysis of five systematic reviews and 37 studies including 22 cohorts concluded that on average, there is a 20% increase in the odds of acquiring cancer in adulthood per standard deviation increase in childhood BMI [5]. These findings imply that prevention or early interventions with obesity in childhood may possibly reduce the rates of cancer in adulthood. However, research also suggests that childhood BMI status alone is not a useful predictor of the independent risk of morbidity in adulthood.

Obesity in childhood and adolescence is concurrent with a state of chronic, low-grade inflammation. Landgraf and colleagues report altered adipose tissue biology, including hypertrophy and hyperplasia in 6–18 years olds with obesity [6]. As adipose tissue expands to contain stores of fat, the microcirculation is disrupted, leading to adipose tissue hypoxia and cell death [7]. Adipose tissue necrosis attracts inflammatory cells and leads to the secretion of pro-inflammatory cytokines such as TNF-a [8]. Several studies have also identified an increased systemic circulation of C-reactive protein (CRP), marking low-grade inflammation as a co-feature in childhood obesity [9–16]. A recent review has described how low-grade inflammation and reduced natural killer cell functionality in obesity may promote malignancy and therefore be a possible causative mechanism for the increased risk of cancer later in life [17]. Furthermore, it is undisputed that chronic inflammation may have detrimental effects on DNA integrity and stability, and that genomic instability—a dynamic state characterised by elevated rates of genetic changes resulting from either cell-cycle dysfunctionalities or events affecting DNA integrity—is an enabling characteristic for the complex, multi-step process of tumorigenesis [18, 19].

In addition, obesity in childhood and adolescence can be associated with a state of micronutrient deficiencies. Deficiencies of several micronutrients including iron, selenium, folate, zinc, and vitamins A, D and E have been identified increasingly in children with obesity [20–24]. However, vitamin D deficiency has attracted the most attention, as it is being diagnosed increasingly in children within the UK [25]. Over the last few years, a large proportion of studies have identified obesity in children as a state of systemic hypovitaminosis D [26–32]. Furthermore, a bi-directional Mendelian randomisation analysis across 42,024 participants has demonstrated a causative association between obesity and vitamin D deficiency, such that a 10% increase in BMI may reduce levels of vitamin D by 4.2% [33]. Several studies report vitamin D to play a role in inhibiting inflammation, protecting cells from DNA damage, inducing cell-cycle arrest and promoting apoptosis [34, 35].

Links between DNA damage and genomic instability with obesity have been previously reported, with well-established, early markers of carcinogenesis found associated with excess adiposity in human participants and animal models [36]. However, so far only a few of these investigations have been conducted in children and adolescents. Higher levels of y-H2AX foci and micronuclei (MNi) were identified in peripheral blood lymphocytes (PBLs) from children with obesity compared to ‘healthy weight’ controls [37, 38]. However, findings of the same type in other tissues were inconclusive. Research in a small cohort of Mexican children did not identify an association between adiposity and nuclear anomalies in the buccal epithelium, yet a more recent study in Italian children found a significant link between childhood obesity and MNi formation in the same tissue [39, 40]. Similarly, there are discrepancies in studies of oxidative DNA damage in children with obesity; one reports positive correlations between obesity status in childhood and serum 8-OHdG, and two reports higher levels of 8-OHdG in urine samples [41–43], while the fourth study of urinary 8-OHdG and BMI in Italian children reports an inverse correlation [44]. Thus, comprehensive analysis of multiple markers of DNA damage and genomic instability, together with markers of systemic inflammation and micro-nutritional deficiencies, may shed light on their combined predictive value and their applicability for the early monitoring of cancer risk in relation to obesity.

To undertake this, we have adopted a non-invasive approach to testing acquired DNA damage, genomic instability, systemic inflammation and vitamin D status alongside multiple markers of adiposity in adolescents recruited from schools and paediatric obesity clinics in London.

## Participants and methods

### Sample size calculation

A cross-sectional study was designed to compare markers of adiposity, inflammation, vitamin D and DNA damage in adolescents aged 10–18 years. The primary endpoint was the frequency of MNi in the buccal mucosa. The required sample size was calculated by extracting data on mean values and variance in adults with normal weight (*n* = 21) and adults with obesity (*n* = 83) from a previous study [45]. These data were entered into G*Power (v3.1) software for A priori calculation of sample size based on a two-tailed, independent means test at an error rate of 1%. The required total sample size was calculated to be 80. Whilst it would have been most appropriate to source data from a study conducted in adolescents, there was a lack of literature reporting mean and variance values for buccal MNi in a cohort of adolescents with obesity. In order to account for this potential source of bias and to cover issues such as missing data, the sample size was increased by 20% with an aim to include a minimum of 96 participants.

### Participant recruitment and screening

Over 200 research packs were distributed across schools in London to pursue non-selective recruitment. Paediatric clinics at St George’s London NHS Trust and King’s College Hospital London supported the recruitment of participants with obesity. In total, four schools and two NHS paediatric obesity clinics agreed to collaborate and 171 participants were screened for inclusion. The screening process required participants to complete a medical questionnaire to be assessed against the following exclusion criteria: dental treatment within last 6 weeks or local inflammation including pain, swelling and other evidence of tooth decay, consumption of medications including multivitamins, X-rays of the head and neck within the last 6 months, medical history of inflammatory conditions, cancer and other general illness (flu, cold, fever) on the day of sample collection. Overall, 132 participants met the inclusion criteria and provided all anthropometric and biological samples.

### Anthropometric assessments

Obesity was defined as >98th percentile of the BMI for age and gender, in accordance with the UK-WHO classification [46]. Participant’s height was recorded using a standard, portable stadiometer (Marsden Weighing Machine Group) to the nearest mm. The TANITA BC54N body composition scales were used to determine weight and body fat percentage via bioelectrical impedance. Participants with a weight >150 kg were analysed for body fat percentage via InBody S10. Waist and hip measurements were recorded to the nearest mm using a standard measuring tape.

### Biological sample collection

For the analysis of vitamin D and CRP, one saliva sample was collected from participants using the Salimetrics Oral Swab (SOS) which was placed on the floor of the oral cavity for one minute. Saliva samples were centrifuged at ×1500*g* and stored at −20 °C until analysis. For the analysis of 8-OHdG, participants collected 10–30 mL of a mid-stream urine sample into a polypropylene universal container. In total, 3 µL of Gentamycin was added per mL of sample and stored at −80 °C until 8-OHdG analysis. For the buccal cytome assay, cell sample collection followed the protocol of Thomas and colleagues with no modifications [47].

### Analysis of inflammation and vitamin D

CRP was quantified in saliva samples using the Salimetrics CRP ELISA kit (Stratech, 1-3302). The analytical sensitivity of the kit was 0.042 pg/mL. Saliva samples were allowed to thaw before they were mixed and centrifuged at ×1500*g* for 15 min at room temperature, in order to remove mucins and particulate matter that can interfere with the assay. Saliva was diluted between 10 to 30-fold and assayed in duplicate. In total, four assays were run to analyse 132 saliva samples. The average *R*-squared value of standard curves was 0.99.

Vitamin D was quantified in saliva samples using the 25-OH Vitamin D (total) ELISA kit (DX-EIA- 5396, Oxford Biosystems). The analytical sensitivity of the kit was 2.89 ng/mL. Saliva samples were allowed to thaw before being mixed and centrifuged at ×1500*g* for 15 min at room temperature. Saliva was not diluted for this assay and samples were analysed in duplicate. In total, four assays were run to analyse 132 saliva samples. The average *R*-squared value of standard curves was 0.96. Seven saliva samples had a vitamin D level below the detection limit of the kit, which led to the exclusion of those participants from the dataset.

### Analysis of DNA damage

The DNA damage EIA Kit (AD-EKS-350, Enzo Life Sciences) was used to perform the quantification of 8-OHdG in urine samples via a competitive ELISA reaction. The analytical sensitivity of the kit was 0.59 ng/mL. Prior to each assay, urine samples were allowed to thaw and centrifuged at ×2000 g for ten min at room temperature. Urine samples were diluted 20–30-fold, vortexed for 10 sec then assayed in duplicate. In total, four assays were run to analyse 132 urine samples. The average *R*-squared value of standard curves was 0.99. To control for intra-individual variation in urinary 8-OHdG levels, a creatinine correction was applied. Urinary creatinine was assessed by the University of Westminster Blood Testing Service (UKAS accredited). Samples were analysed in the ILab Aries based on the colorimetric methodology between the reaction of creatinine with picric acid under alkaline conditions. Urinary creatinine (mg/mL) was calculated by multiplying the concentration of creatinine (mmol/L) in urine samples by the molecular weight of creatinine then divided by one hundred. Final 8-OHdG (ng/mg creatinine) was calculated by dividing urinary 8-OHdG (ng/mL) by urinary creatinine (mg/mL).

### Analysis of genomic instability by buccal cytome assay

The buccal cytome assay (BCA) was performed within one week of buccal cell sample collection according to the protocol published by Thomas et al. with some modifications [47]. Cell sample homogenisation was performed using a 25 G needle. In total, 150 µL of cell sample was added to two cytocentrifuge cups. Slides were then centrifuged at 600RPM for five min at room temperature and allowed to air dry for ten min. The slide staining process followed that of Thomas et al. [47]. Slides were examined under a Carl Zeiss Primo Star Light Microscope (37081). Unsatisfactory slides (low cell counts, debris obscuring cells or poor staining) were repeated. To score slides, the microscope was connected to a JENOPTIK ProgRes CT5 USB C Camera (D-07739 Jena) and images were captured using ProgRes Software. One thousand cells per participant were imaged at a magnification of ×1000 with immersion oil. Slides were scored blindly for their frequency of normal differentiated cells (NDCs), cells with MNi, polynucleated cells (PNCs) and cells with nuclear buds/nucleoplasmic bridges (NBUDs/NPBs). The frequency of each cell type was reported as per 1000 cells.

### Statistical analysis

Data were entered into BMI SPSS version 25 and checked for outliers using boxplots. Extreme outliers (20 out of 132) were removed from subsequent analysis. Unpaired *t*-tests were conducted on anthropometric and biomarker data of the remaining 112 participants to test for any differences between participants with normal range BMI and those with obesity. Levene’s test for equality of variances was applied. The three genomic variables, Buccal MNi, Buccal PNCs, and Buccal NBUDs/NPBs, were combined to create a new variable: combined genomic instability score or combined GI score.

Pearson’s correlations were conducted on all variables to identify relationships. Testing for a false discovery rate was carried out to manage the risk of a Type 1 error. Two multiple linear regression models were conducted with a combined genomic instability score as the criterion variable. The first was a hierarchical linear regression analysis in which all predictor variables were included in the model: demographic variables were included in the first stage and the remaining variables in the second stage. A multiple linear regression analysis was also conducted in which only those variables that correlated with the criterion variable were included. The following assumption tests were conducted: multicollinearity, homoskedasticity, normality of errors and linearity, resulting in the removal of predictor variables: waist circumference and fat percentage.

### Ethical considerations and approvals

Ethical approval was obtained from the Human Research Authority and NHS Research Ethics committee (IRAS ID: 212869) and the University of Westminster (ETH1617-1943). Data were protected in accordance with the Data Protection Act 1998 and later updated according to the General Data Protection Regulation and Data Protection Act 2018. Written consent was obtained from the parents of all participants that were screened and included in the study. Biological samples were stored in line with the Human Tissue Act 2004.

## Results

### Participant demographics

A contingency analysis of participants’ demographics was performed relative to the BMI category (Table 1). Fifty-nine participants were identified as with normal range BMI (<98th centile) and 53 participants were identified with obesity (>98th centile). In total, the largest proportion of participants were White British or White European (38%) and the smallest proportion was of mixed parentage (6.7%). A Chi-squared analysis revealed no significant differences in ethnic groups across BMI categories (*p* = 0.39). There was a fairly equal distribution of males and females across BMI categories (F: M ratio of 1: 0.84). The average age of participants across categories was 13.6 years in the normal range BMI group and 14.6 years in the group with obesity. Overall, the age range of participants—10–18 years—was the same within categories.

**Table 1:** Participant demographics by BMI category.

	Normal range BMI	Obese	Total
*Ethnicity*			
White British or White European	19	24	43
Black or Black British	16	12	28
South Asian or South Asian British	14	6	20
Arab or Arab British	6	7	13
Mixed parentage	4	4	8
*Sex*			
Female	32	29	61
Male	27	24	51
*Age*			
Age mean (SD)	13.63 (2.36)	14.60 (2.05)	
Age range (years)	10–18	10–18	
*Total*	59	53	112

### Obesity is associated with higher levels of genomic instability in the buccal mucosa

**Fig. 1:**
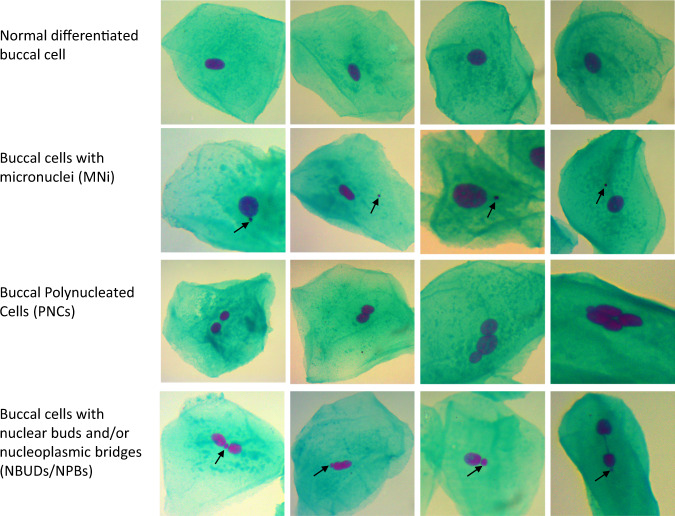
Nuclear anomalies in buccal cells from participants. Photomicrographs of exfoliated buccal mucosa cells stained with Feulgen and Light Green and viewed at 1000× magnification under transmitted light. The figure shows examples of normal differentiated cells (top row) compared to cells presenting different types of nuclear abnormalities (rows below) (buccal MNi = cells with micronuclei; PNCs = cells showing poly-nucleation or multiple nuclei; NBUDs/NPBs = cells with nuclear buds and/or nucleoplasmic bridges). Cells were scored and nuclear abnormalities classified according to the criteria defined in the ‘buccal micronucleus cytome assay’ [45].

A comparison of participants’ anthropometric and biomarker measures is reported in Table 2. As expected, the average BMI Z-score (the standard deviation above or below the mean), waist to hip ratio (WHR), waist circumference and body fat percentage were all significantly higher for participants in the group with obesity (>98th centile). Unpaired *t*-test analysis of average levels of biomarkers between normal range BMI and participants with obesity revealed significantly higher levels of buccal cells with MNi, polynucleated buccal cells (PNCs), and nuclear buds and nucleoplasmic bridges (NBUDs/NPBs) (Fig. 1). Participants with obesity presented with higher levels of CRP and oxidative DNA damage (8-OHdG) in urine, and lower levels of vitamin D in saliva. However, those differences were not statistically significant.

**Table 2:** Comparisons of Participants’ anthropometric and biomarker measures.

	BMI category	*N*	Mean	SD	*t*	*p*
Z score	Normal range	59	0.31	0.94	17.66	<0.001
	Obese	53	3.35	0.88		
WHR	Normal range	59	0.81	0.07	5.43	<0.001
	Obese	53	0.91	0.11		
Waist circumference (mm)	Normal range	59	700.02	69.35	10.27	<0.001
	Obese	53	1092.06	271.63		
Body fat (%)	Normal range	59	23.12	6.79	14.08	<0.001
	Obese	53	42.37	7.50		
CRP (pg/mL)	Normal range	59	1842.46	1205.74	1.83	0.100
	Obese	53	2262.12	1465.51		
Vitamin D (ng/mL)	Normal range	59	7.64	3.65	1.70	0.093
	Obese	53	6.29	5.12		
8-OHdG (ng/mg creatinine)	Normal range	59	186.58	109.36	1.93	0.057
	Obese	53	238.12	158.60		
Buccal MNi (%)	Normal range	59	1.03	1.00	5.06	<0.001
	Obese	53	2.09	1.24		
Buccal PNCs (%)	Normal range	59	7.27	3.46	3.20	0.002
	Obese	53	9.45	3.90		
Buccal NBUDs/NPBs (%)	Normal range	59	1.05	1.36	5.27	<0.001
	Obese	53	2.83	2.16		
Combined genomic instability score (%)	Normal range	59	0.94	0.43	5.86	<0.001
	Obese	53	1.44	0.50

### Adiposity is correlated with levels of inflammation, vitamin D, acquired DNA damage and genomic instability

**Fig. 2:**
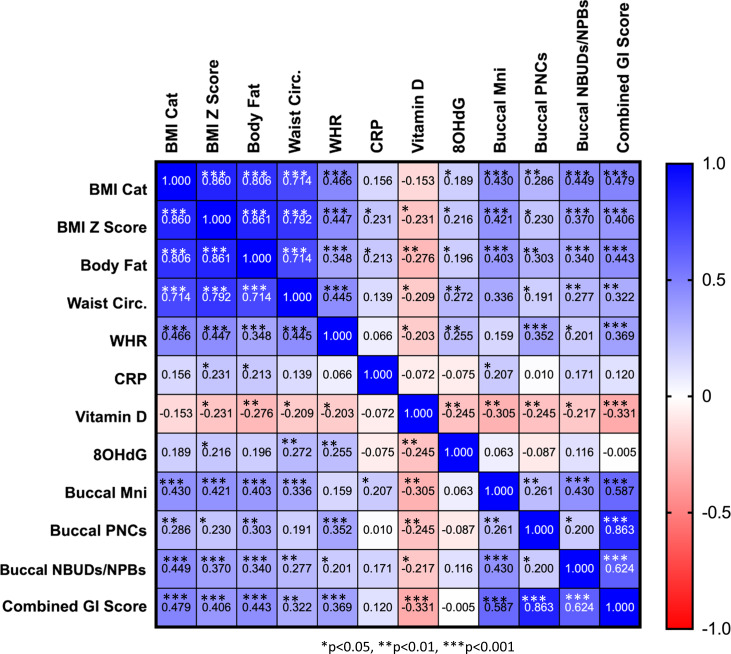
Pearson’s correlation coefficients of biomarkers across all participants. Abbreviations used in the map: BMI cat body mass index category, WHR waist-hip-ratio, CRP C-reactive protein, 8OHdG 8-hydroxy-2′-deoxyguanosine, Buccal Mni buccal micronuclei, Buccal PNCs buccal polynucleated cells, Buccal NBUDSs buccal nuclear buds, Combined GI score combined genomic instability score.

Pearson’s correlation analysis of biomarkers across all participants revealed a statistically significant correlation between all anthropometric markers (*p* < 0.001). BMI Z-score and body fat percentage are markers of adiposity that were positively correlated with CRP, urinary 8-OHdG and genomic instability markers (Fig. 2). Furthermore, BMI Z-score and body fat percentage were negatively correlated with salivary vitamin D. WHR and waist circumference were also negatively correlated with vitamin D and positively correlated with DNA damage and genomic instability markers, except that WHR was not correlated with the frequency of MNi. However, neither WHR nor waist circumference correlated with levels of inflammation. Interestingly, salivary CRP correlated positively with levels of cells with MNi (*p* = 0.029) and salivary vitamin D correlated negatively with all DNA damage and genomic instability markers.

### BMI category, vitamin D and oxidative DNA damage are predictors of genomic instability (models 1 and 2)

The hierarchical multiple regression analysis in Model 1, with BMI category, age, sex and ethnicity as the predictors, explained 21.2% of the variance and was significant, F (7, 104) = 5.28, *p* < .001. Model 2 in which Vitamin D, CRP, 8-OHdG and waist-hip ratio were added explained significantly more variance (*R*^2^ change = 0.11, F (4, 100) = 4.21, *p* = 0.003). Model 2 explains 29.9% of the variance (adjusted *R*^2^ = 0.299) and was significant (F (11, 100) = 5.30, *p* < 0.001). The significant predictors in model 2 were BMI category, 8OHdG and vitamin D as indicated in Table 3a.

**Table 3:** Unstandardised and standardised regression coefficients for the variables entered into (a) models 1, 2 and (b) model 3.

(a) Models 1 and 2						
Model		*B*	Std. error	*β*	*t*	*p*
1	BMI category	0.555	0.093	0.529	5.99	<0.001
	Age (years)	−0.033	0.021	−0.142	−1.58	0.116
	South Asian or South Asian British	0.150	0.131	0.110	1.15	0.254
	Sex	0.086	0.094	0.082	0.91	0.365
	Arab or Arab British	−0.082	0.149	−0.050	−0.55	0.581
	Mixed Parentage	−0.055	0.190	−0.027	−0.29	0.773
	Black or Black British	−0.006	0.114	−0.005	−0.05	0.960
2	BMI category	0.449	0.104	0.428	4.32	<0.001
	Vitamin D (ng/mL)	−0.031	0.010	−0.264	−3.07	0.003
	8-OHdG (ng/mL creatinine)	−0.001	0.000	−0.193	−2.22	0.029
	WHR	0.959	0.538	0.181	1.78	0.078
	Age (years)	−0.028	0.020	−0.121	−1.41	0.163
	South Asian or South Asian British	0.119	0.124	0.087	0.96	0.342
	Sex	0.080	0.095	0.076	0.84	0.404
	CRP	7.921E−6	0.000	0.025	0.31	0.761
	Black or Black British	0.034	0.111	0.028	0.30	0.764
	Mixed Parentage	0.001	0.182	0.001	0.01	0.994
	Arab or Arab British	−2.309E−5	0.149	0.000	0.00	0.999

(b) Model 3					
Variable	*B*	SE B	*β*	*t*	*p*
BMI category	0.487	0.085	0.464	5.72	<0.001
Vitamin D (ng/mL)	−0.036	0.010	−0.301	−3.66	<0.001
8-OHdG (ng/mL) creatinine	−0.001	0.000	−0.166	−2.01	0.047

### Multiple regression analysis (model 3)

Those variables that were significant predictors in model 2 were entered into a multiple linear regression using the standard method. A significant model emerged: *F* (3, 108) = 17.14, *p* < 0.001. The model explains 30.4% of the variance in the combined genomic instability score (adjusted *R*^2^ = 0.304). Table 3b gives information about regression coefficients for the predictor variables entered into the model showing that BMI category, Vitamin D and 8-OHdG were significant predictors. Therefore, participants’ predicted combined genomic instability score is equal to 0.841 + 0.487 (BMI category) −0.036 (Vitamin D) −0.001 (8-OHdG), where BMI was coded as 1 = normal range, 2 = obese.

## Discussion

This research is the first to perform a combined, non-invasive investigation of inflammation, vitamin D, and multiple markers of genome damage in relation to anthropometric measurements in adolescence. Our results support the hypothesis that childhood obesity is associated with increased genomic instability and presents implications for a potential increase in the risk of cancer later in life. Most importantly, we have presented for the first time a multiple regression model for the prediction of genomic instability based on obesity, urinary 8-OHdG and salivary vitamin D.

We found that BMI Z-score and body fat percentage significantly and positively correlated with salivary CRP levels, supporting the hypothesis that low-grade inflammation may increase with adiposity. Although average salivary CRP levels were higher in participants with obesity, this was not statistically significant due to large variations across both cohorts. Several studies have found significantly higher serum CRP from children with obesity compared to controls [9–16, 48], but results in saliva are so far limited and inconclusive. Naidoo and colleagues reported a BMI above the 85th centile to be a significant predictor of elevated salivary CRP across 170 Black South African children [49]. Salivary CRP levels were also reported to be six times higher in children with a BMI above the 95th centile [50]. However, more recently Janem and colleagues reported no significant differences in salivary CRP between children with and without obesity in a study of 49 participants [51]. Nonetheless, moderate correlations between serum and salivary CRP in adolescents indicate that excess production of serum proteins may drive them to become incorporated into saliva [52]. Therefore, further studies would be useful to explore the predictive potential of inflammatory markers in saliva, in relation to cancer risk.

Furthermore, we found the frequency of cells with MNi, a type of biomarker of genomic instability, increased in correlation with salivary CRP levels. There are several indications that describe inflammation to have a causative role in the initiation of malignancy. For example, a one-unit increase in CRP levels has been linked with a 2.29-fold increase in the risk of colon cancer [53]. Chronic inflammation is a well-known aetiological factor for genetic instability and neoplastic transformations in cells and may drive the production of excess reactive oxygen and nitrogen species and cause structural modifications in DNA to promote pre-malignancy [54, 55]. A profile of circulating pro-inflammatory cytokines has been associated with pre-malignant lesions of the oral mucosa, gastric mucosa and prostate in the absence of tissue infection [56–59]. There are also reports that chronic inflammation may suppress anti-tumour defence mechanisms, lead to a loss of mitotic arrest following DNA damage and enable the accumulation of random mutations that may contribute to the genetic heterogeneity seen in cancer cells [60–62]. These indications coupled with the associations between excess adiposity and inflammation fuel the need for long-term monitoring of inflammation status in patients with obesity.

In addition, we found an increase in multiple markers of adiposity correlated with a significant decrease in salivary vitamin D levels, and that average vitamin D was lower in participants with obesity, although this was not statistically significant. Over the last few years, a large proportion of studies have identified obesity in children as a state of hypovitaminosis D [26–32]. To our knowledge, this is the first study to explore the association between adiposity in adolescents and vitamin D levels in saliva. The lack of investigations of this type so far may be due to the challenges associated with saliva as a dilute biological fluid compared to serum. Therefore, the detection of vitamin D in saliva from patients with deficient serum levels requires a high sensitivity assay. For this study, the commercially available immunoassay kit with the highest analytical sensitivity was sourced. We also obtained saliva samples using the stimulated method of saliva collection as this has been demonstrated to increase levels of salivary vitamin D [63]. However, there were seven samples that were below the detection limit of the assay, and it is important to note that six of these samples were from participants with obesity. Therefore, there is a need for the development of high-sensitive and high-throughput assays that can quantify vitamin D in saliva and be used to develop a threshold for point of care diagnosis of a deficiency, especially since a strong correlation between salivary vitamin D and serum vitamin D (*R* = 0.83) has been reported [64].

Most importantly, we found that salivary vitamin D may be a useful predictor of genomic instability when combined with weight status and urinary 8-OHdG levels via a multiple regression model. It is likely that deficient levels of vitamin D in obesity may exacerbate the effects of adipose tissue dysfunction and consequent DNA damage. Unlike this investigation, previous studies have not assessed vitamin D status and DNA damage simultaneously in childhood obesity. However, excess DNA damage in sperm cells has been related to vitamin D deficiency and excess adiposity in a rat model [65]. Associations between vitamin D and markers of genomic integrity and stability have been presented before and align with our findings. Sufficient serum vitamin D status has been found to modulate the effects of UV-light induced MNi formation in human lymphocytes [66]. Furthermore, vitamin D treatment has also demonstrated a reduction in MNi frequency of rat hepatocytes and in a model of murine lymphoma [67]. There are indications that vitamin D may reduce oxidative DNA damage and up-regulate DNA repair proteins to preserve genomic integrity [68–70, 68, 69]. Several mechanisms could explain links between vitamin D and reduced oxidative DNA damage. Vitamin D has been described as a hormone with anti-inflammatory properties by a number of studies [34, 71–73]. Although we did not find a correlation between salivary CRP and vitamin D, it is of interest that previous research has found vitamin D deficiency in childhood obesity to coincide with increased serum high-sensitivity CRP levels [23]. Studies in rodents imply that vitamin D deficiency can increase the secretion of pro-inflammatory cytokines [74, 75]. Overall, it can be postulated that vitamin D deficiency is a modifiable risk factor for genomic instability in children with obesity.

Another important finding is that 8-OHdG—as a measure of DNA damage—correlated significantly and positively with multiple markers of adiposity in our cohort of participants, but did not differ significantly between participants with and without obesity. To date, there are only three other investigations that we are aware of which have assessed the association between adiposity and levels of 8-OHdG in urine. An earlier report demonstrated children with obesity to have higher levels of 8-OHdG in urine [43]. However, an inverse association between BMI and urinary 8-OHdG was reported across 159 healthy Italian children [44]. Yet more recently, higher levels of urinary 8-OHdG were reported in children with obesity, corroborating the earlier findings, but these children also presented with insulin resistance [42]. Our findings support the associations between adiposity and oxidative DNA damage, similar to the findings of El Wakkad et al. [41], who reported body fat percentage assessed via bioelectrical impedance and BMI to be positively correlated with 8-OHdG in serum across 103 adolescents. These findings are important because of the role that oxidative DNA damage can play in generating genomic instability and a pre-cancerous state.

There is evidence that oxidative DNA damage can promote genomic instability. 8-OHdG can contribute to chromosomal instability by altering the maintenance of telomere length [76]. A recent review has highlighted that 8-OHdG lesions occur more frequently in telomeric DNA than bulk genomic DNA or microsatellite regions [77]. This is interesting because telomere length in children with obesity has been found to be 23% shorter compared to age-matched healthy weight controls [78]. Telomere shortening is also associated with an increase in nuclear anomalies, in particular nuclear buds and bridges [79]. These findings stress the importance of including levels of 8-OHdG to predict chromosomal instability in childhood obesity.

It is of interest that we found average levels of three different nuclear anomalies in the buccal mucosa to be significantly higher in participants with obesity compared to healthy weight controls. To the best of our knowledge, our research is the first to present significant correlations between multiple markers of adiposity and the frequency of biomarkers of genomic instability such as micronucleated cells, nuclear buds/bridges and PNCs in adolescence. In 2009, the first investigation of adiposity and nuclear anomalies in the buccal mucosa found no association but was conducted with only 20 Mexican 7–11 years olds in each weight category [39]. More recently, Idolo and colleagues investigated the impact of various lifestyle factors on nuclear anomalies in the buccal mucosa of 6–8 years old Italians and concluded obesity in children to be an independent risk factor for increased MNi frequency [40]. There is evidence that an increased prevalence of MNi in the oral mucosa may be reflective of chromosomal instability occurring in other tissues [80]. Therefore, it is unsurprising that our findings support previous research that found an increased level of MNi and chromosomal aberrations in PBLs from children with obesity [37]. Overall, these findings confer that childhood obesity is associated with an increased state of acquired DNA damage and genomic instability. Therefore, it is important to consider the potential implications of our findings in relation to cancer risk.

An increased frequency of MNi in buccal epithelial cells has been consistently identified in pre-malignancy and malignancy of the aerodigestive tract [81–85]. There is also evidence to suggest that an increased MNi frequency in buccal cells may be related to the risk of cancer at other sites including the breast, uterus, lung, colorectum, and bladder [86–89]. There are indications that MNi containing whole chromosomes can proceed into several cell generations and be reincorporated into the genome following further mitotic divisions [90]. MNi division cycles can lead to catastrophic genetic re-arrangements in a single or few chromosomes—a newly described mutational process called chromothripsis [91]. Such localised chromosomal re-arrangements may be transferred to daughter nuclei in subsequent mitotic cycles and play a role in generating a pre-cancerous genome. Furthermore, MNi can display a lack of nuclear envelope integrity when occurring in cancer cells [92]. Firstly, impaired nuclear envelope function has been related to an increase in DNA damage in MNi within cancer cells—a process that may also promote chromothripsis [92]. It is also likely that the nuclear envelope of a micronucleus is more likely to rupture, causing exposure of self-DNA to the cytosol. Possible immuno-stimulatory consequences of this event have recently been reported in a mouse model and human cancer cells [93]. This means that the occurrence of MNi may also drive carcinogenesis by triggering inflammation. Interestingly, we found a significant correlation in the oral cavity between levels of CRP and cells with MNi across all participants. Longitudinal studies in large cohorts may consolidate the use of the buccal cytome assay as a tool for cancer risk prediction.

Also important are the findings of excess average levels of nuclear buds and nucleoplasmic bridges in participants with obesity compared to controls. NBUDs/NPBs has been described as a consequence of unrepaired DNA damage or gene amplification and have also been linked with carcinogenesis. An increased frequency of nuclear buds and nucleoplasmic bridges was documented in PBLs from cancer patients compared to healthy controls [89]. Furthermore, nucleoplasmic bridges are associated with breakage-fusion-bridge (BFB) cycles that may drive chromosomal rearrangements seen in tumour genomes [94]. DNA amplification and chromosomal rearrangements have been noted in cancers of the lung, breast, prostate, GI tract and skin [95].

We also found a higher average frequency of PNCs in participants with obesity, which is indicative of increased cytokinesis failure. Cancer cell lines have been used to demonstrate the mutagenic consequences of binucleated cells [96]. In a recent review, tetraploidy and cytokinesis failure have been evaluated as mechanisms for aneuploidy in subsequent mitotic cycles. It has been suggested that these events can lead to genetic diversification in cancer cells that possibly provide developmental advantages [97]. An increased frequency of binucleated cells has been identified in patients with cancers of the breast, head, neck and mesothelium [98–100]. Furthermore, a number of different mitotic and cell cycle checkpoint proteins that regulate cytokinesis can be mutated in cancer, indicating that cytokinesis failure could possibly be an early event in tumorigenesis [101].

In summary, our results support the hypothesis that childhood obesity is associated with increased genomic instability. Importantly, we have found that obesity, vitamin D and oxidative DNA damage can together predict genomic instability. This investigation has several strengths. Firstly, we combined analysis of multiple markers of DNA damage and genomic instability which have been linked to the initiation and progression of cancer. Secondly, we investigated several parameters of adiposity in addition to traditionally reported BMI Z-scores in children and adolescents. Furthermore, we have outlined a novel, non-invasive approach for combined assessment of inflammation, micronutrition and genome health. Our findings warrant further research into the applicability of this approach as a non-invasive clinical tool for predicting early, pre-pathological genomic changes in young patients with obesity.

## Conclusions

By applying a non-invasive approach for the combined assessment of parameters of adiposity, inflammation, Vitamin D status and genome damage, we have developed a multiple regression model for the prediction of genomic instability in adolescence. Further work may lead to its application in the prioritisation and provision of clinical intervention measures to prevent increased risk of malignancy in patients with obesity.
